# A Rare Case of Angiolymphoid Hyperplasia With Eosinophilia With a New Effective Treatment

**DOI:** 10.7759/cureus.39966

**Published:** 2023-06-05

**Authors:** Unaiza Hasan, Najia Ahmed, Tariq Malik, Syed Arbab Shah, Uroosa Subhan

**Affiliations:** 1 Dermatology, Pakistan Navy Station (PNS) Shifa Hospital, Karachi, PAK; 2 Dermatology, Bahria University of Health Sciences, Pakistan Navy Station (PNS) Shifa Hospital, Karachi, PAK

**Keywords:** external auditory canal, therapy, propranolol, adrenergic beta antagonists, angiolymphoid hyperplasia with eosinophilia

## Abstract

Angiolymphoid hyperplasia with eosinophilia (ALHE) is a benign locally proliferating lesion of unknown etiology, composed of vascular channels lined by endothelial cells, surrounded by lymphocytes and eosinophils. It presents clinically as a cluster of skin to violaceous-colored nodules on the head and neck, particularly in and around the ear. We present the case of a 50-year-old, Pakistani woman with unilateral multiple nodular lesions for eight years in the left ear concha and postauricular area causing complete obliteration of the external auditory meatus with conductive hearing loss of the left ear for seven years. Biopsy showed lymphoid follicles and dilated blood vessels with mixed infiltrate predominantly eosinophils corresponding to the diagnosis of angiolymphoid hyperplasia with eosinophilia. Surgical excision was not feasible, and there was no response to topical steroids. The patient was started on beta blockers. After three months, postauricular lesions completely resolved, and the size of the rest of the nodules decreased markedly; then hearing loss also recovered. Our objective in this study is to emphasize the importance of considering beta blockers for the treatment of ALHE.

## Introduction

Angiolymphoid hyperplasia with eosinophilia (ALHE) is a benign tumor in which blood vessel proliferation occurs along with dense eosinophilic infiltrate [1]. The frequency of this disease is not known, but cases have been reported worldwide, being more common in Japan. It can persist for years with no malignant transformation being reported so far. ALHE is more common in females, although male predominance in selected Asian studies is also reported. It mostly affects middle-aged adults. ALHE clinically presents in the skin as violaceous-colored pruritic nodules and papules. Common sites include the ear and the periauricular area [1,2]. It can rarely present on the extremities, trunk, and genitalia. ALHE may be asymptomatic, or the patient may complain of pruritus, pain, or bleeding [3]. Diagnosis is confirmed by skin biopsy for histopathology which shows proliferation of blood vessels that are lined by epithelioid endothelial cells with prominent perivascular infiltrate mainly eosinophils. Treatment of ALHE is quite challenging due to a high recurrence rate. Surgical excision is common. Other treatment modalities like pulsed dye laser, carbon dioxide laser, oral retinoids, and topical/systemic corticosteroids are recommended, but spontaneous resolution has been reported [3,4]. However, the treatment of ALHE with oral beta blockers is quite promising. Within three months of oral propranolol, the lesions improved with no recurrence. Additionally, no obvious adverse effects of oral beta blockers were observed after one year of treatment [5]. In this report, we describe a case of ALHE diagnosed in a 50-year-old female who presented with multiple nodular lesions in and around the left ear. Herein, we explain the importance of beta blockers for the treatment of ALHE, as our patient showed excellent response to them. 

## Case presentation

**Figure 1:**
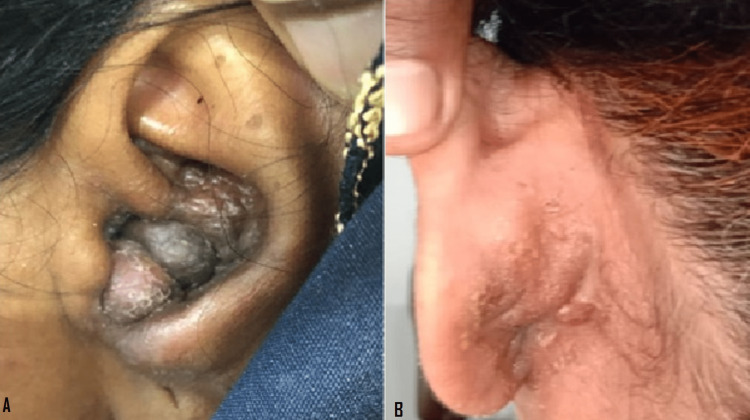
Erythematous to violaceous-colored, nodular lesions in the left concha, causing complete obliteration of the external auditory meatus (A) and skin-colored papules and nodules can be seen in the postauricular region (B).

A 50-year-old, Pakistani woman presented with eight years history of multiple nodular lesions in and around the left ear, associated with pruritis and bleeding upon scratching, and decreased hearing in the affected ear. It was not associated with pain, headache, discharge, fever, night sweats, or weight loss. Nodules were initially single and small and rapidly increased in size and number during the first year followed by a gradual increase in size over seven years. No aggravating and relieving factors were known. Prior to these lesions, there was no history of recent trauma or infection. On examination, unilateral multiple erythematous to violaceous-colored, round to oval, papular, and nodular lesions in the left concha and postauricular area were seen causing complete obliteration of the external auditory meatus (Figure 1). Few lesions were dome-shaped. They were variable in size, and the largest one was 1.5 x 2cm, with a rough and slightly scaly surface, soft in consistency, and the lesions were attached to the underlying surface. They were non-tender and non-pulsatile with normal temperature. The rest of the mucocutaneous examination was unremarkable. On ENT examination, the Weber test was performed, and the sound lateralized toward the left ear; using the Rennie test, bone conduction was found to be greater than air conduction, which confirmed conductive hearing loss of the left ear.

**Figure 2:**
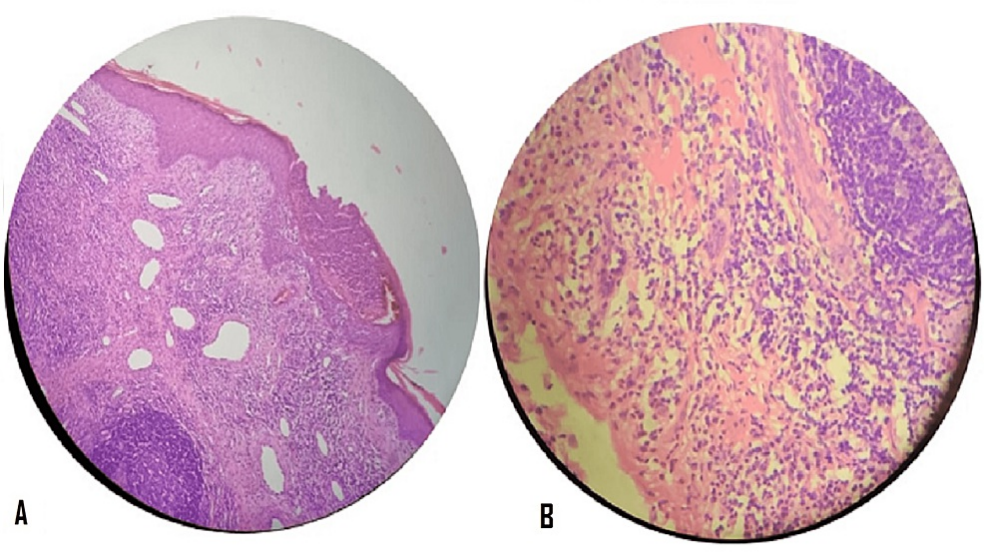
H&E-stain slide on 10x magnification (A) shows small- and medium-sized dilated blood vessels and lymphoid follicles with prominent germinal centers; 40x magnification (B) shows mixed infiltrate predominantly eosinophils.

Laboratory investigations, including complete blood count, thyroid profile, liver and renal function test, urine examination, and fasting blood sugar, were normal. ECG and echocardiography were normal. CT scan of the left temporal bone showed otomastoiditis. Incisional biopsy from postauricular nodules was done for histopathological examination which showed subepithelial lymphoid follicles with prominent germinal centers. There were dilated blood vessels and mixed infiltrate predominantly eosinophils with foci of hemorrhage (Figure 2). There was no evidence of granuloma or malignancy.

**Figure 3:**
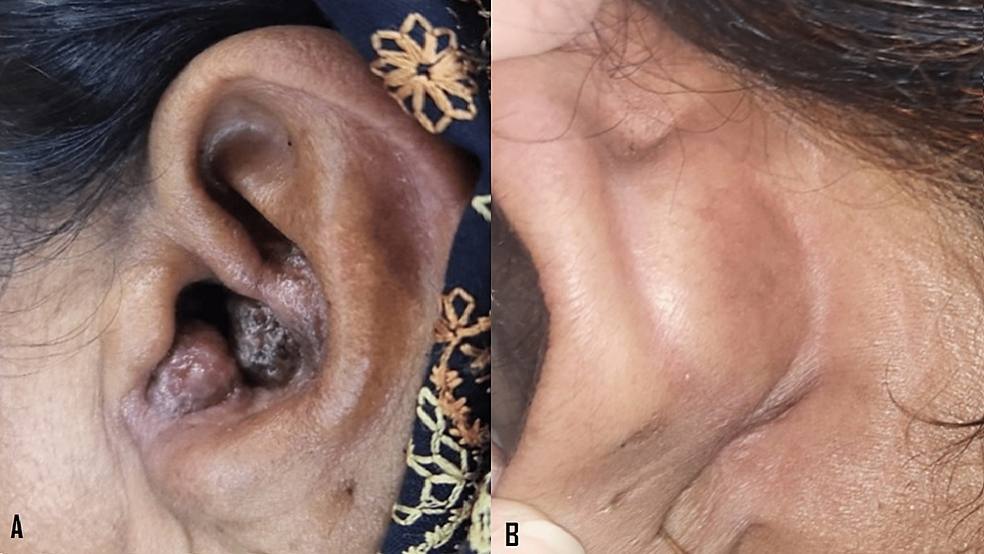
After three months being on beta blockers, the nodule size decreased markedly, and hearing loss due to obliteration of the external auditory meatus recovered (A); postauricular lesions completely resolved (B).

The ENT surgeon did not consider surgical excision feasible for this patient, and she was already on topical super potent steroids for two years, with no response. After monitoring vital signs and ECG and ruling out COPD, the patient was started on beta blocker, 1 mg/Kg/day (half tablet propranolol 40 mg three times a day) for the first week, followed by 2 mg/kg/day (tablet propranolol 40mg three times a day) with regular monitoring of vital signs and ECG. After three months being on the beta blockers, postauricular lesions completely resolved, and the size of the rest of the nodules decreased markedly. Then hearing loss due to obliteration of the external auditory meatus recovered (Figure 3).

## Discussion

ALHE presents as a cluster of benign cutaneous lesions most commonly in the head and neck. It may bleed (25%) in addition to causing pruritis (37%) or pain (20%) [6]. In our case, lesions were present in clusters unilaterally in the left ear, in accordance with the majority of the literature reviews being most common in the head and neck, and were associated with pruritis and bleeding but were not associated with pain. Twenty percent of cases of ALHE exhibit peripheral eosinophilia [6]. However, in our patient, the blood count showed normal eosinophils. ALHE is characterized by a florid proliferation of blood vessels lined by plump endothelial cells and admixed with a dense inflammatory infiltrate of lymphocytes, eosinophils, and mast cells [7]. Likewise in our case, histopathology showed similar changes, that is, dilated blood vessels with lymphoid follicular aggregates with admixed infiltrate predominantly eosinophils and lymphocytes. Which confirmed the diagnosis of ALHE. Surgical excision appears to be the most effective treatment for ALHE [3]. However, surgical excision was not feasible in our case as advised by the ENT surgeon. And many other therapeutic approaches are available to treat ALHE; However, recurrence rates have been noted to be high for surgical excision (40.8%), pulsed dye laser (50%), and carbon dioxide laser (54.6%). This analysis managed to emphasize again the inefficiency of medical treatments with elevated recurrence rates using oral isotretinoin (100%) and corticosteroids (87.8%) [3]. In contrast, our patient showed no response to topical steroids, but a good response to beta blockers.

A single sitting of intralesional radiofrequency ablation led to almost complete resolution of ALHE of the ear with no recurrence at three-month follow-up [8]. However, we did not have this option available in our hospital to implement it on our patient.

There were two successful treatments of ALHE with oral propranolol: the first case was a 56-year-old female with slowly enlarging papulonodular lesions on the retroarticular area and the front of her scalp for two years; upon eight weeks of treatment with oral propranolol 40mg/day, retroarticular lesions disappeared completely, along with significant regression of the other lesions [5]. Likewise, our patient with ALHE was treated with oral propranolol. Her retroarticular lesions resolved completely, with good regression of others.

Our patient is on regular follow-up with us. She has been on beta blockers for six months now. Lesions are successfully regressing with no new lesion formation. Additionally, the drug is well tolerated with no adverse effects observed so far.

## Conclusions

ALHE is a rare benign disease, malignant transformation is very rare. For treatment of ALHE, beta-blockers should be considered standard treatment provided they are beneficial being suitable, safe, cost-effective, easily available, and well-tolerated drugs with excellent treatment response for ALHE.
